# The Role of the Key Differentially Mutated Gene *FGFR3* in the Immune Microenvironment of Bladder Cancer

**DOI:** 10.1155/2022/7952706

**Published:** 2022-08-12

**Authors:** Jun Jiang, Yan Zhan, Jun Li

**Affiliations:** Department of Oncology, The Central Hospital of Wuhan, Tongji Medical College, Huazhong University of Science and Technology, Wuhan 430014, China

## Abstract

The tumor microenvironment (TME) has been a major focus of research in recent years as a crucial factor in the development and progression of bladder cancer. Unfortunately, the precise composition of TME, particularly the immunological and stromal components, remains unknown. In this work, we downloaded the RNA-seq expression profiles and somatic mutation data of 433 bladder cancer cases from The Cancer Genome Atlas (TCGA) and then employed a comprehensive bioinformatics approach to evaluate them. Firstly, the expression profiles were used to predict the scores and then the content of immune and stromal cells via the estimate package in R software. We then identified differentially expressed genes (DEGs) and differentially mutated genes (DMGs) according to the high-stromal score cohort and low-stromal score cohort. Finally, fibroblast growth factor receptor 3 (FGFR3) was the main differentially mutated gene in bladder carcinoma that we discovered after conducting a cross-study on DEGs and DMGs. Follow-up investigation revealed that *FGFR3*, whose expression correlated inversely with cancer progression stage, appeared to be a protective factor in bladder cancer. The method of Gene Set Enrichment Analysis (GSEA) was employed to, respectively, interpret the expression data of FGFR3 in high and low expression lists. We observed that the genes in the low FGFR3 expression list were strongly enriched in the biological processes associated with transplantation and cell adhesion, suggesting the possible role of *FGFR3* in predicting TME metastasis status in bladder cancer. Therefore, this study is aimed at investigating whether *FGFR3* is promising as a biomarker of TME remodeling to explain underlying mechanisms involved in tumorigenesis and metastasis, which may help to make decisions on treatments for bladder cancer.

## 1. Introduction

Bladder tumor, the most common type of which is urothelial carcinoma, usually begins in umbrella cells in the bladder lumen. Urothelial tumors can be classified as bladder tumors (accounting for about 90% to 95% [1] of urothelial tumors), upper urinary tract tumors, and proximal urethral tumors. Bladder tumor, which mainly consists of noninvasive and invasive tumors [2], is highly prevalent in men and less prevalent in women. Approximately 1.1 percent of men and 0.27 percent of women will develop bladder cancer in their lifetime [3]. It was expected that 500,000 new cases of bladder cancer and 200,000 deaths from bladder cancer would be seen worldwide in 2020 [4, 5]. The detection and treatment of advanced and localized diseases are the main topics of current research on the genetics and molecular biology of bladder tumors. Treatments for different types of bladder cancers vary. The main treatment for nonmuscle invasive bladder cancer is intravenous BCG (Bacillus Calmette-Guérin) vaccination, while options for advanced cancer and muscle-invasive bladder cancer are more diversified, including checkpoint inhibitors (such as PD-L1/PD1 and CTLA-4), targeted therapies (such as CAR T cell therapy), and immunotherapy using antibody-drug conjugates [6–8]. Although treatments for bladder cancer using antibody-based anti-PD-L1/PD1 and anti-CTLA-4 have shown to be clinically effective, they cannot currently satisfy the treatment needs of all patients, as most patients have exhibited a poor response to immunotherapy at various stages of bladder cancer. Therefore, a better knowledge of immunotherapy resistance mechanisms may point to the right path for better bladder cancer treatment.

Immunotherapy has been successful in bladder cancer treatment, but there is still a need to improve the efficiency of response and predictability of patient response after treatment, as tumors can escape immune detection through a variety of mechanisms, such as immunosuppression and the formation of a tolerant TME [9]. Bladder tumor is related to malignant changes in a variety of cells and structures, such as epithelial cells, vascular cells, and extracellular matrix. Efficient antitumor immune responses and immune-mediated tumor clearance require the joint efforts of antigen-presenting cells, lymphocytes, and natural killer cells. Tumors, on the other hand, would release numerous immunosuppressive and antiapoptotic substances for survival and impede the normal activities of these immune cells, such as IL-6, PGE2, IL-10, and TGF-*β* [9–12], resulting in a highly tolerant TME. Furthermore, TME is closely associated with the accumulation of immune cells with an immunosuppressive phenotype, like Marrow-Derived Suppressor Cells (MDSC), tolerogenic DCs (tDC), and Tumor-Associated Macrophages (TAM). A highly immunosuppressive microenvironment features an elevated expression of PD-L1, abundant MDSC and TAM, increased production of PGE2, and abnormal metabolism of glycosaminoglycans (such as hyaluronan). The above studies have shown that there is heterogeneity in immune composition and immune response of bladder cancer patients and these heterogeneities may have a significant impact on patients' final clinical outcomes. Though research on TME has produced useful findings, little work has been done on the biological characteristics and role of TME in bladder cancer patients, so it is necessary to make an in-depth genetic analysis to explore the dynamic transition of TME, helping to illustrate the underlying mechanisms of bladder cancer development.

In our study, we analyzed DEGs and DMGs across the stromal composition of bladder cancer cases and found that *FGFR3* has the potential to be an immune-related predictive biomarker. Besides, we may reveal the mechanisms behind tumorigenesis and bladder tumor progression, hoping to throw light on the treatment of bladder cancer.

## 2. Data and Methods

### 2.1. Raw Data

The TCGA database was used to acquire the somatic mutation data and RNA-seq expression profiles of 433 bladder cancer cases (414 tumor samples; 19 normal samples) and the clinical data of 412 patients with bladder carcinoma.

### 2.2. Prognostic Values of Immune Score, Stromal Score, and Estimate Score

The immune score (presenting the level of infiltrating immune cells in tumor tissue), stromal score (presenting the level of stroma in tumor tissue), and estimate score (the sum of immune and stromal scores) were calculated for each bladder cancer sample utilizing the estimate package in R software. A higher score represents a higher fraction of the corresponding component (immune cells, stroma, or overall components) in TME.

### 2.3. Survival Analysis

408 tumor samples were screened from 433 bladder cancer cases concerning the following conditions: (i) remove nontumor samples; (ii) remove samples that do not contain clinical information. The survminer and survival packages in R software were used to perform the survival analysis. The Kaplan-Meier method was adopted to plot survival curves. Statistical significance was determined by the log-rank test, and the *p* value significance level was set at 0.05.

### 2.4. Correlation Analysis of Clinicopathological Features and Scores

We conducted a correlation analysis of all scores with clinicopathological features using the ggpubr package in R software. The Wilcoxon rank-sum test or the Kruskal-Wallis rank-sum test was carried out to determine statistical significance.

### 2.5. Identification of DEGs in the High- or Low-Stromal Score Group

We analyzed samples from the group with high- or low-stromal scores using the limma package in R software and identified DEGs. DEGs meeting the following criteria were considered as significantly differential genes: (i) log2 fold change (FC) (high-stromal score cohort/low-stromal score cohort) with an absolute value greater than 1; (ii) false discovery rate (FDR) < 0.05.

### 2.6. Somatic Mutation Analysis and Identification of DMGs in the High- or Low-Stromal Score Group

We extracted somatic mutation data for bladder tumors from the database TCGA and saved the data in Mutation Annotation Format (MAF). A total of 414 tumor samples were divided into high-stromal or low-stromal cohorts according to the middle value of the stromal score. We compared the high- and low-stromal cohorts via the maftools package in R software to identify DMGs [13], and a *p* value less than 0.05 was considered statistically significant.

### 2.7. Enrichment Analysis of DEGs by Gene Ontology (GO) and Kyoto Encyclopedia of Genes and Genomes (KEGG)

To explore the biological functions and signaling pathways of DEGs, GO and KEGG gene enrichment analyses were conducted on 1823 DEGs utilizing clusterProfiler, enrichplot, and ggplot2 packages in R software. Enrichment results that meet the following criteria were judged as being significant: (i) *q* value < 0.05; (ii) *p* value < 0.05. The pheatmap package was used to create DEG heatmaps.

### 2.8. Gene Set Enrichment Analysis

The C2.CP.KEGG gene set was obtained from the Molecular Signature Database (MSigDB), and the clusterProfiler package in R was used to conduct GSEA.

### 2.9. Cell Culture

We obtained three bladder cell lines (RT-112, T24, and 5637) and a normal human bladder epithelial cell line (SV-HUC-1) from BeNa Culture Collection (BNCC, China). RT-112 and 5637 cell lines were cultured in RPMI-1640 medium (Gibco, USA), T24 cell line in McCoy's 5a medium (Gibco, USA), and SV-HUC-1 cell line in F-12K medium. All the cells were cultured with 10% FBS (Gibco, USA) and the condition of 5% CO_2_ and 37°C.

### 2.10. qRT-PCR

Total RNA was isolated by TRIzol (Invitrogen, USA) and measured by the NanoDrop 2000 (Thermo Fisher Scientific, USA). 1 *μ*g RNA was reversely transcribed into cDNA via the PrimScript RT Reagent Kit (Takara, Japan). Next, the qRT-PCR was conducted via the SYBR-Green Master Mix (Life Technologies, USA) under Agilent MX3000. The 2−*ΔΔ*Ct method was employed to determine FGFR# relative expression with the internal control GAPDH. The sequences of *FGFR3* primers are as follow: forward, 5′-AACACAGTGGAGCGAATTCCTTT-3′ and reverse, 5′-GCACGGTAACGTAGGGTGTG-3′.

## 3. Results

### 3.1. Research Workflow

**Figure 1:**
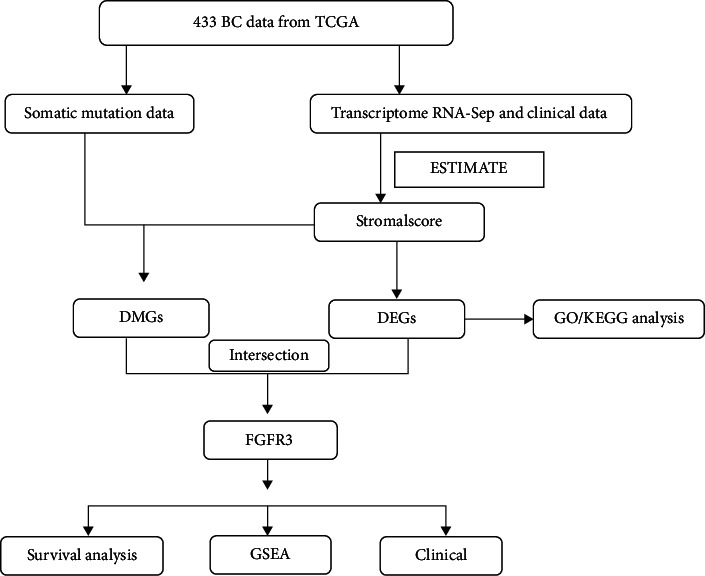
Workflow of this research.

The research workflow is presented as follows (Figure 1). The estimate method was adopted to generate scores separately for immune component and stromal component in 433 bladder cancer cases after RNA-seq expression profiles, and relevant clinical information had been obtained from the database TCGA. We then downloaded somatic mutation data and grouped the cases based on the median of stromal component scores. Meanwhile, we detected DMGs between high- and low-stromal score groups and conducted GO and KEGG analyses on the DEGs. Then, DEGs and DMGs were intersected and five key genes were obtained—AKAP6, NLRP7, NRP2, *FGFR3*, and MAP4K1. Focusing on *FGFR3*, we further carried out a correlation analysis to explore the relation of *FGFR3* to overall survival and clinicopathological characteristics and GSEA.

### 3.2. Clinicopathological Features of Bladder Cancer Patients from TCGA

RNA-seq expression profiles and clinical data of 433 bladder cancer cases were obtained from the TCGA. Among them, the data of 408 bladder cancer patients meet the experimental needs, and their clinicopathological characteristics are summarized in Table 1.

### 3.3. Identification of DEGs in Bladder Cancer Patients

**Figure 2:**
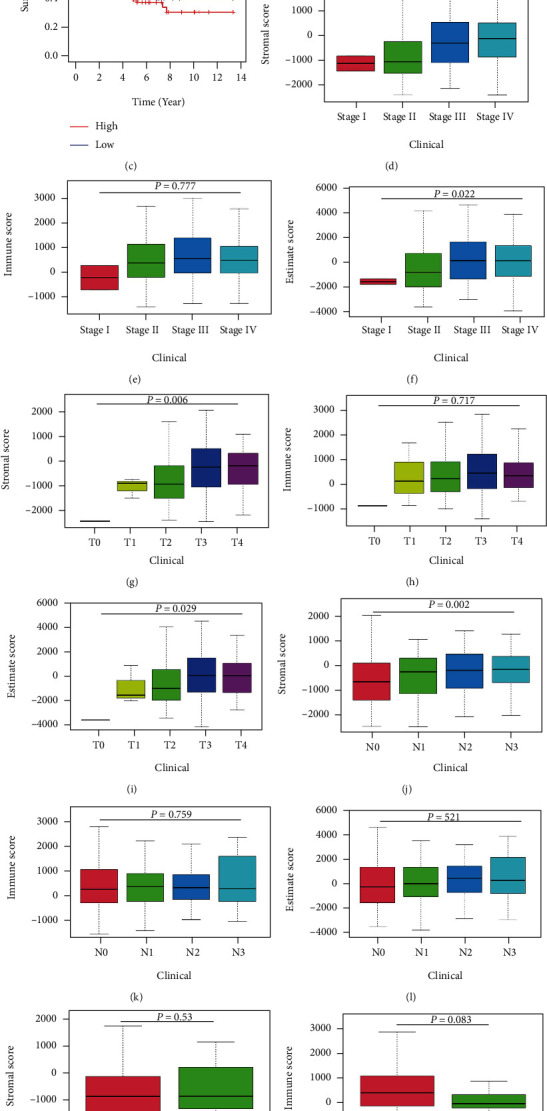
Correlation analysis of immune score and stromal score with survival and clinicopathological characteristics in bladder cancer cases. (a–c) Kaplan-Meier survival analysis of bladder cancer cases by comparing them with the median to determine high and low scores in immune score, stromal score, and estimate score, which was tested by the log-rank test. (d–f) Distribution of immune score, stromal score, and estimate score at different stages of cancer, which was tested by the Kruskal-Wallis rank-sum test. (g–i) Distribution of the three scores in T staging, which was tested by the Kruskal-Wallis rank-sum test. (j–l) Distribution of the three scores in N staging, which was tested by the Kruskal-Wallis rank-sum test. (m–o) Distribution of the three scores in M staging, which was tested by the Wilcoxon rank-sum test.

Patients were classified into high- and low-score groups based on the median of the three scores after calculating the immune score, stromal score, and overall score, and then, survival analysis on the high- and low-score groups was separately conducted. The association of the three scores with the overall survival (OS) of patients was revealed (Figures 2(a)–2(c)). Among the three scores, the stromal score appeared to show a more negative correlation with OS (*p* = 0.125), suggesting the greater potential of the stromal score to predict the prognosis of bladder cancer patients. The clinical data of bladder cancer cases were then analyzed to investigate the association of the three scores with clinicopathology (Figures 2(d)–2(o)). Stromal scores were found to be significantly diverse at different tumor stages and different TNM stages. The above findings illustrate that the stromal component is crucial to bladder cancer progression, especially to tumor invasion and metastasis.

### 3.4. Identification of DEGs Based on Stromal Scores

**Figure 3:**
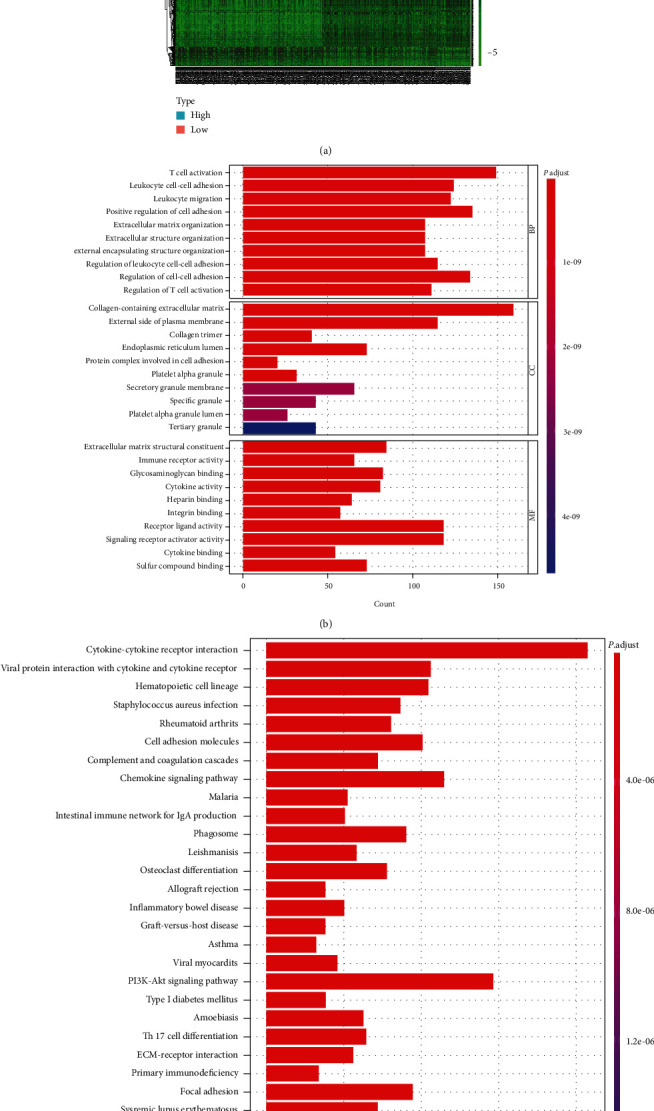
Heatmap, GO, and KEGG enrichment analysis. (a) DEG heatmap generated by comparing high and low subgroups of stromal score. The row names of the heatmap are the gene names, and the column names are sample IDs. The Wilcoxon rank-sum test was conducted to determine DEGs, and FDR < 0.05 and ∣log2FC | >1 were considered significant. (b, c) GO and KEGG enrichment analyses of 1823 differential genes, of which result with *p* < 0.05 and *q* < 0.05, were considered significant.

Because of the greater potential of stromal scores to assess the prognosis of bladder cancer, we performed differential expression analysis on two stromal score groups. We obtained 1,823 DEGs with 1524 genes being upregulated and 299 genes being downregulated. The heatmap of differential genes is shown (Figure 3(a)). In addition, GO enrichment analysis results showed that the functions of DEGs were associated with the regulation of cell adhesion and the adhesion function of cells (Figure 3(b)). Moreover, the KEGG enrichment analysis revealed that DEGs were highly enriched in biological processes related to cell adhesion, such as cytokine-cytokine receptor interactions and cell adhesion molecules (Figure 3(c)). The above results indicate that cell adhesion-related biological processes may represent the main function of DEGs. Since cell adhesion is essential to tumor cell metastasis, the stromal component is the important composition of TME in bladder cancer patients.

### 3.5. Identification of DMGs Based on Stromal Scores

**Figure 4:**
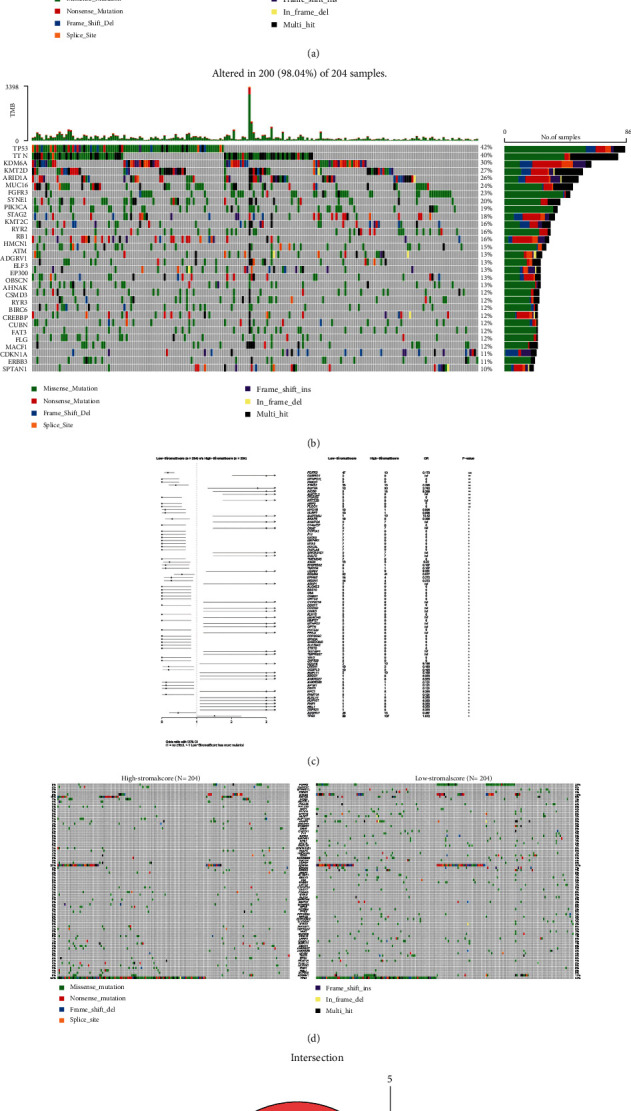
Analysis of somatic mutations between the two immunity groups and identification of common genes in DEGs and DMGs. (a, b) Waterfall plots showing the distribution of the top 30 most frequently mutated genes. (c) Forest plot showing significantly differentially mutated genes between the two cohorts with ^∗∗∗^*p* < 0.001, ^∗∗^*p* < 0.01, and ^∗^*p* < 0.05. (d) Waterfall plot showing 80 DMGs between the high- and low-immunity groups. (e) Venn diagram showing the intersecting genes between DEGs and DMGs.

There is growing evidence that tumor mutations can generate specific neoantigens that activate immune recognition and thus kill tumor cells [14–16]. To ascertain the relationship between the stromal component in TME and gene mutation, we explored whether there were genetic differences between the two stromal cohorts based on the median stromal scores. We analyzed and visualized the somatic mutation data from these two cohorts and found the top 30 most frequently mutated genes (Figures 4(a) and 4(b)). The proportion of mutated genes in the low-stromal cohort was larger than those in the high-stromal cohort. There were 80 DMGs between the two cohorts (Figures 4(c) and 4(d)). In addition, there were five intersecting genes between DEGs and DMGs, namely, *AKAP6*, *NLRP7*, *NRP2*, *FGFR3*, and *MAP4K1* (Figure 4). The mutation frequency of *FGFR3* was lower in the high-stromal group than that in the low-stromal group, suggesting that the smaller the mutation frequency of *FGFR3*, there may be more tumor stromal cells, thus making the tumor more prone to metastasis.

### 3.6. FGFR3 Expression as Well as Survival and Clinicopathological Characteristics of Bladder Cancer Cases

**Figure 5:**
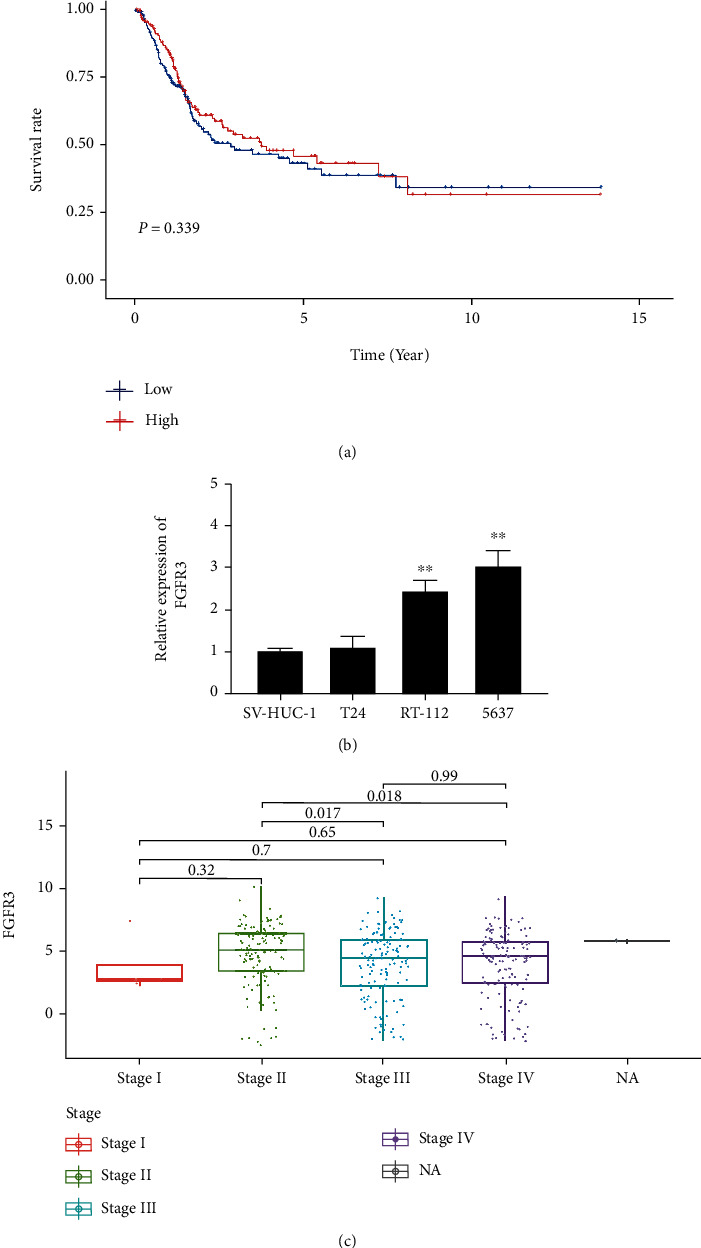
Differential expression of *FGFR3* and its correlation with survival time and clinicopathological characteristics of bladder cancer. (a) Survival analysis of bladder cancer patients with different *FGFR3* expression levels. Patients were classified into groups with high or low *FGFR3* expression levels by comparing the median *FGFR3* expression level. It was tested by a rank-sum test with *p* = 0.339. (b) *FGFR3* with high expression in RT-112 and 5637 cells. ^∗∗^*p* < 0.01 vs. SV-HUC-1. (c) Correlation of *FGFR3* expression with clinicopathological features. The Kruskal-Wallis rank-sum test was used for statistical significance testing.

In our research, we classified bladder cancer samples into a high *FGFR3* expression group and a low *FGFR3* expression group according to the median *FGFR3* expression. Survival analysis of the two groups revealed that no significant difference was found in OS of bladder cancer patients between the two groups (Figure 5(a)). Furthermore, compared with SV-HUC-1 cells, the expression of *FGFR3* was upregulated in RT-112 and 5637 cells (Figure 5(b)). We analyzed *FGFR3* in combination with clinicopathological features and found that *FGFR3* expression increased and then decreased gradually with the advancement of the tumor stage (Figure 5(c)). Taken together, *FGFR3* expression, which is related to TME remodeling and tumor metastasis, is a relevant marker of tumor metastasis in bladder cancer.

### 3.7. FGFR3 May Be a Target for Remodeling TME

**Figure 6:**
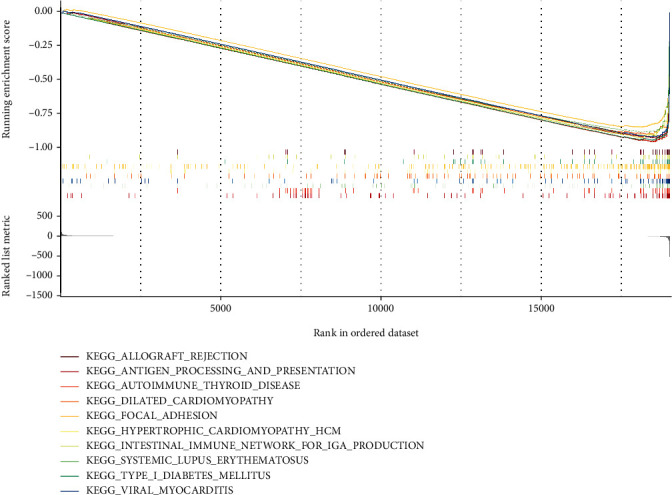
GSEA on samples with low *FGFR3* expression and the enrichment of low *FGFR3* expression in the C2 collection.

Based on the aforementioned findings, we inferred that *FGFR3* expression was not significantly associated with OS, but was correlated with clinicopathological features. We performed GSEA on high and low *FGFR3* expression cohorts. For the C2 collection in MSigDB, *FGFR3* genes of low expression were predominantly enriched in biological processes correlated with transplantation and cell adhesion, such as allograft rejection, and the focal adhesion pathway (Figure 6). The above results suggest that *FGFR3* may be a promising target for identifying different TME states in bladder cancer.

### 3.8. Relationship between FGFR3 and Immune Cell Infiltration

**Figure 7:**
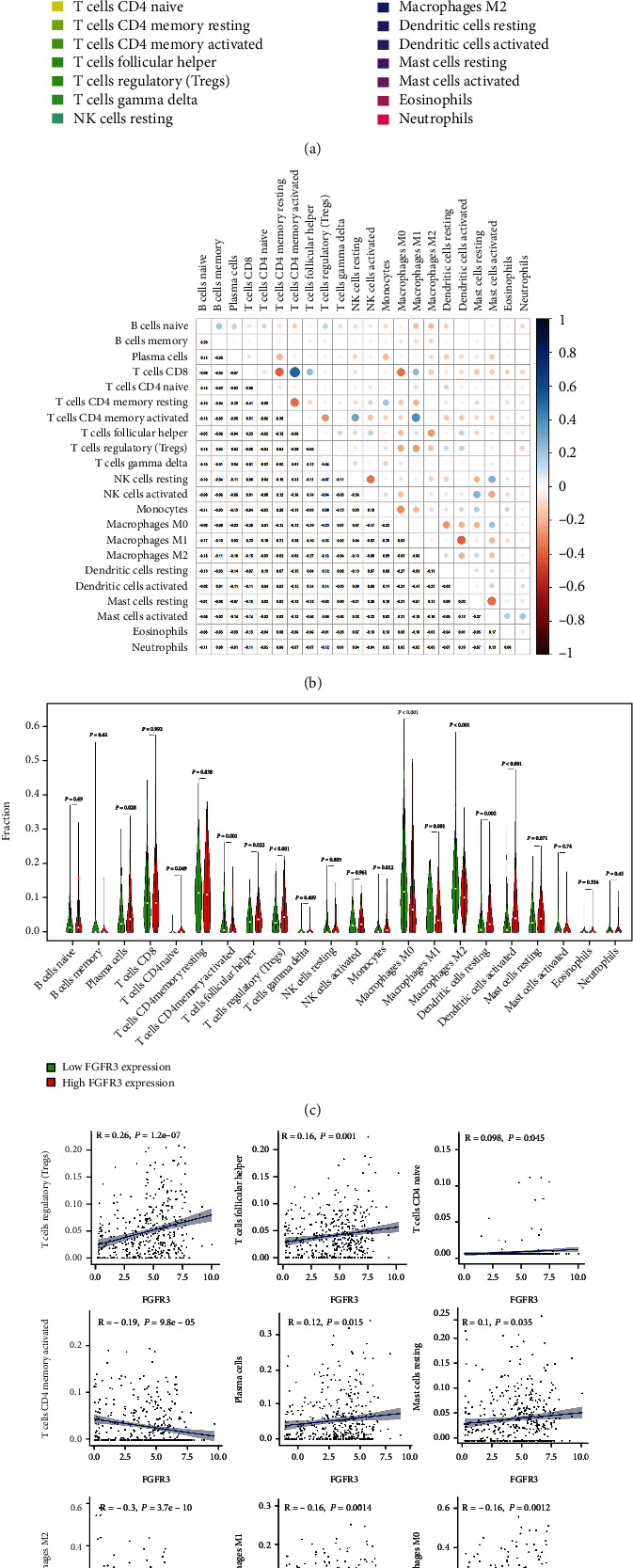
Relationship between FGFR3 and immune cell infiltration. (a) The proportion of infiltrated immune cells in bladder cancer patients; (b) heatmap of the correlation between immune cells; (c) changes in the median ratio of 22 infiltrated immune cells in bladder cancer samples from the high *FGFR3* expression group and low *FGFR3* expression group; (d) the association between the variation trend of the proportion of 11 kinds of immune cells and *FGFR3* expression.

We established an expression profile of 22 immune cells in the bladder cancer samples using the CIBERSORT algorithm, so as to investigate the impact of *FGFR3* on immune cell infiltration. Based on median *FGFR3* expression, we divided all bladder cancer samples into the high-expression group and low-expression group for further analysis of the difference in infiltrated immune cells between the two groups. Finally, 11 immune cells with significant differences were identified. Accordingly, a figure, expressing the relationship between the gene expression and the concentration of immunological cells in each sample, was drawn to further clarify the association between *FGFR3* expression level and the number of immune cells (Figure 7).

### 3.9. Relationship between FGFR3 and Common Immune Checkpoints

**Figure 8:**
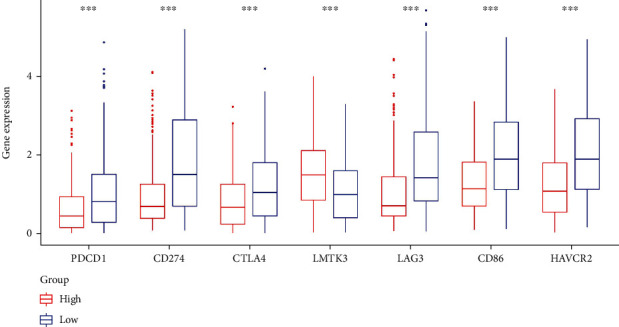
Relationship between *FGFR3* and common immune checkpoints.

For assessing the immunotherapy response of *FGFR3*, we investigated the relationship between *FGFR3* expression and such immune checkpoints as PDCD1, CD274, CLTA4, and LMTK3. The result showed the expression of immune checkpoint genes was generally higher in the low expression group, suggesting that patients with low *FGFR3* expression tended to have a better response to immunotherapy (Figure 8).

We planned to utilize a bioinformatics technique to find predictive immune-related biomarkers in bladder cancer TME by downloading RNA-seq expression data and somatic mutation data from The Cancer Genome Atlas (TCGA). First, we computed the scores of immune and stromal components of the RNA-seq data of bladder cancer patients via the ESTIMATE algorithm, and the results showed that stromal components had greater potential to predict the overall survival (OS) of bladder cancer patients. So, we performed a further analysis based on high- and low-stromal scores. By comparing the somatic mutation data of the high- and low-stromal score groups, we discovered that there were considerable differences in gene mutation levels between these two groups. Based on the above work, we identified 1823 differentially expressed genes (DEGs) and 80 differentially mutated genes (DMGs). In the intersection of DEGs and DMGs, we found the stroma-related biomarker—fibroblast growth factor receptor 3 (*FGFR3*). The tyrosine kinase receptor *FGFR3*, encoded by the FGFR3 gene located on chromosome 4, plays a crucial role in bone formation, osteogenesis, and maintenance [17, 18]. It is now recognized that the *FGFR3* signaling pathway overlaps with some oncogenic pathways in the human body, including the *EGFR/RAS/PI3K/ERK/AKT* pathway, and is correlated with the epithelial-to-mesenchymal transition of tumor [19, 20]. The *FGFR3* gene may affect tumorigenesis even before gene translation: the circRNA product of FGFR3 gene transcription—has_circ_0068871 is overexpressed in bladder tumors and linked to tumor cell proliferation and migration [21]. The above findings suggest that *FGFR3* may be crucial in the TME of bladder cancer. Therefore, we further analyzed *FGFR3*-related biological properties utilizing Gene Set Enrichment Analysis (GSEA), of which the outcomes indicated that *FGFR3* is an immune-related predictive biomarker that may be important in different TME states of patients with bladder carcinoma. In this study, we started with the analysis of DEGs and DMGs generated from the comparison of the stromal composition of bladder cancer cases and found that *FGFR3* has the potential to be an immune-related predictive biomarker. Besides, we may reveal the fundamental mechanisms of tumorigenesis and the progression of bladder cancer, which is expected to contribute to the improvement of bladder cancer treatment.

## 4. Discussion

The goal of this research is to find important genes with different mutations and expressions in the TME of bladder cancer patients to better understand their prognosis. A series of comprehensive bioinformatic analyses finally showed that *FGFR3* met our expectations. We believe that *FGFR3* is important to the biological function of tumor metastasis and could be a promising target for remodeling TME.

TME is vital in the incidence and progression of cancer. It has been found to be effective to change TME from tumor-friendly to tumor-suppressive as a means of enhancing cancer therapy [22]. TME is the complex environment that surrounds the tumor, including epithelial cells, infiltrating immune cells, and extracellular matrix. These structures influence tumor growth, dissemination, and immune tolerance. Large amounts of immunosuppressive cytokines can be released by tumor cells and infiltrating cells in the TME to promote tumor growth by preventing T cell proliferation and effector function [23, 24]. Although bladder cancer has been rated as the most prevalent disease in the urinary system, the association between TME and tmotehe prognosis of bladder cancer has been poorly understood [25]. Therefore, it is necessary to further investigate it.

This research shows that the stromal component in TME seems to have an important role in the clinical endpoints related to patients with bladder cancer. TME influences survival rate and shows differences across cancer stages, suggesting that the stromal component is strongly associated with bladder cancer invasiveness and distant metastasis. Additionally, evidence has revealed that tumor mutations can generate specific neoantigens that activate immune recognition and kill tumor cells, suggesting that TME status may be affected by modifications to specific genes [26, 27]. As a result, exploring new targets in TME to increase bladder cancer immunotherapy is a new challenge for bladder cancer treatment. Although many previous articles have studied gene expression profiles or somatic mutation data, little work has been done on the underlying mechanisms in TME of bladder cancer. Here, we performed a comprehensive bioinformatic analysis utilizing transcriptome RNA-seq data and somatic mutation data and we found that the decreased expression of *FGFR3* correlates with tumor metastasis. The study also showed that *FGFR3* was more prone to mutations in the low stromal group, and these mutations may generate tumor-specific neoantigens in bladder cancer. According to GSEA, the biological process involving tumor cell metastasis was highly linked with the group with low FGFR3 expression. Hence, this study suggests that *FGFR3* may be a potential target that can influence survival and immunotherapy response and remodel TME in bladder cancer patients.

*FGFR3* encodes a member of the FGFR family. Members of the FGFR family vary from each other in tissue distribution and ligand affinities. An extracellular portion exists in a full-length representative protein, and it contains three immunoglobulin-like domains, a cytoplasmic tyrosine kinase domain, and a single hydrophobic membrane-spanning segment. The extracellular region interacts with fibroblast growth factors to trigger a series of downstream signals that ultimately impact mitogenesis and differentiation (provided by RefSeq, Aug 2017) [28].

The FGFR proteins are engaged in a cascade of pathways associated with cancer. These receptors, when activated, can deactivate the RAS-MAPK, PI3K-AKT pathways, etc. The mechanisms of misregulated FGFR vary among cancers. In lung and breast cancers, receptor amplification has been observed. Numerous malignancies including coding mutations and deletions have been observed in multiple cancers, and more recently, FGFR fusions that activate the pathway have been proved to have carcinogenic potential in a variety of cancers. The effectiveness of the targeted therapies ponatinib, dovitinib, and pazopanib in treating overactive FGFR signaling has led to the development of diagnostic sequencing that targets the FGFR genes, particularly in patients with lung cancer [28–30].

In this study, the results of the integrated bioinformatical analysis suggested FGFR3 as a possible indicator for TME status remodeling and clinical outcomes such as distant metastasis, overall survival rate, and immunotherapy response to bladder cancer. Fundamental mechanisms of the immunobiological process associated with FGFR3 need to be further explored to throw light on the treatment of bladder cancer [31].

## 5. Conclusion

Utilizing comprehensive bioinformatic analysis, we found *FGFR3* as a target for TME remodeling status and possibly as a predictor of clinical outcomes, including survival of bladder cancer patients, distant tumor metastasis, and immunotherapy response. Thereby, future research should concentrate on determining the precision of comprehensive *FGFR3* expression analysis. Furthermore, the *FGFR3* target must be evaluated in fundamental experiments as well as clinical trials.

## Figures and Tables

**Table 1:** Statistics of clinicopathological characteristics of bladder cancer cases.

	Overall
*n*	408
Futime (mean (SD))	757.33 (813.00)
Fustat = 1 (%)	157 (38.5)
Age (mean (SD))	68.08 (10.61)
Gender = male (%)	301 (73.8)
Grade (%)	
High grade	384 (94.1)
Low grade	21 (5.1)
Unknown	3 (0.7)
Stage (%)	
Stage I	2 (0.5)
Stage II	130 (31.9)
Stage III	140 (34.3)
Stage IV	134 (32.8)
Unknown	2 (0.5)
T (%)	
T0	1 (0.2)
T1	3 (0.7)
T2	38 (9.3)
T2a	25 (6.1)
T2b	56 (13.7)
T3	43 (10.5)
T3a	70 (17.2)
T3b	81 (19.9)
T4	10 (2.5)
T4a	43 (10.5)
T4b	5 (1.2)
TX	1 (0.2)
Unknown	32 (7.8)
M (%)	
M0	196 (48.0)
M1	11 (2.7)
MX	198 (48.5)
Unknown	3 (0.7)
N (%)	
N0	237 (58.1)
N1	46 (11.3)
N2	75 (18.4)
N3	8 (2.0)
NX	36 (8.8)
Unknown	6 (1.5)

## Data Availability

Data sharing is not applicable to this article as no datasets were generated or analyzed during the current study.
